# The Spectrum of Clinical Presentation of Multiple Sclerosis and Neuromyelitis Optica Spectrum Disorder in Young Patients of Our Community

**DOI:** 10.7759/cureus.19576

**Published:** 2021-11-14

**Authors:** Hikmatullah K Sherani, Mohammad Hasan, Hassan Mumtaz

**Affiliations:** 1 Neurology, Pak-Emirates Military Hospital Rawalpindi, Rawalpindi , PAK; 2 House Officer, Jinnah Postgraduate Medical Centre, Karachi, PAK; 3 Medicine, Jinnah Sindh Medical University, Karachi, PAK; 4 Public Health, Health Services Academy, Islamabad, PAK; 5 Forensic Medicine, Riphah International University, Islamabad, PAK; 6 General Medicine, Surrey Docks Health Center, London, GBR; 7 Urology, Guy's & St Thomas Hospital, London, GBR

**Keywords:** transverse myelitis, optic neuropathy, opticospinal, rion, aquaporin-4, neuromyelitis optica, multiple sclerosis, clinical profile

## Abstract

Background

Neuromyelitis optica spectrum disorder (NMOSD) is an inflammatory, demyelinating syndrome of the central nervous system (CNS) which affects the spinal cord and optic nerves. The aim of our study was to evaluate the clinical appearance of multiple sclerosis and neuromyelitis optica spectrum disease in young children in our community

Materials and methods

This cross-sectional study was done in the neurology departments of the Combined Military Hospital & Pak Emirates Military Hospital Rawalpindi for six months from April 2020 to September 2020. Eighty people between the ages of 18 and 45 with Guillain-Barre syndrome (GBS), multiple sclerosis (MS), and neuromyelitis optica spectrum disorder participated in this prospective research. The patient's demographic profile includes information such as age, gender, length and kind of sickness, and symptoms. The relationship of socio-demographic factors was assessed with the involvement of more than one organ system at the time of presentation.

Results

The final study comprised of 80 patients of whom 31 (38.5 percent) were men and 49 (61.25%) were women. Fifty-four (67.5%) patients were diagnosed with multiple sclerosis while 26 (32.5%) had neuromyelitis optica spectrum disorders. Most of the patients presented with sensory symptoms followed by visual disturbances. Twenty-nine (37%) had involvement of more than one system while 51 (63%) had involvement of only one system at the time of presentation. Female gender and having a diagnosis of multiple sclerosis had a statistically significant relationship with involvement of one than one system.

Conclusions

In individuals with MS and NMO, motor, sensory, and visual symptoms were often observed at the time of presentation. Involvement of one than one system at the time of presentation was fairly common among these patients and females and patients having a diagnosis of multiple sclerosis were more at risk of involvement of more than one system.

## Introduction

Multiple sclerosis (MS) and neuromyelitis optica spectrum disorder (NMO-SD) have become more frequent diagnoses in Pakistan in the past decade owing to advancements in neurology and an increase in the number of neurologists training in Pakistan [1,2]. Prevalence of these disorders has been similar in other parts of the world including western countries [3]. Multiple etiological models have been proposed for these disorders, but the immune-based model has been widely accepted and also remains the basis for most of the management options for these chronic clinical conditions [4].

The main challenge for early diagnosis for these illnesses includes the variety of symptoms with which the patients may present. These symptoms and presentation patterns vary to an extent that some patients may present with optical symptoms alone and others may present with isolated paresis of any limb or bladder problems [5]. This complexity of presentation makes it difficult both for the patient and clinicians to understand the disease phenomenon and reach the facility where they could be managed best. General physicians, emergency doctors, psychiatrists, and medical teams should have a clear idea of various presentations of these illnesses to refer these patients to the neurology team as early as possible [6].

Studies have been done in both western countries and Pakistan to look for the various clinical presentations these patients may present with to the health care facility. In 2016, Jarius et al. published a study on German patients and came up with the findings that female predominance was found in NMO-SD patients. Blindness in one eye was noted in more than 70% of the patients. Around 40% of the patients had a significant disability which resulted in impairment of the patients suffering from this immunological disorder. Response to steroids was promising among these patients [7]. In 2014, Barhate et al. performed a study in India and came up with the findings that neuromyelitis optica spectrum disorders were far more prevalent among female patients as compared to male patients with a ratio of 7:1. All patients included in their study had spinal cord lesions and around 20% had lesions in the brain, consistent with lesions of multiple sclerosis [8]. Nazish et al. did a similar study in the Kingdom of Saudi Arabia and concluded that patients with multiple sclerosis had optic neuritis and myelitis as common clinical presentations. Sensory system involvement was seen in more than 70% of the patients, followed by motor and visual symptoms [9].

Neurology and neuro-radiology have been in the evolving phase in Pakistan and neurology doctors, internal medicine doctors, and general physicians should have a fair idea of the spectrum of symptoms with which these patients may present. Early diagnosis and referral to appropriate service may reduce the disability among these patients and improve quality of life. They found that NMO-SD accounts for more than half of central nervous system demyelinating disorders in Pakistan, where it is often misdiagnosed for multiple sclerosis, according to a study published in 2019. Accurate diagnosis is critical since certain MS medications have the potential to exacerbate NMOSD [10]. The reason for doing this research was to examine how multiple sclerosis and neuromyelitis optica spectrum disease present clinically in young individuals in our area.

## Materials and methods

We conducted a cross-sectional study in the neurology departments of the Pak Emirates Military Hospital Rawalpindi and the Combined Military Hospital Rawalpindi for six months, from April 2020 to September 2020. Ethical approval was obtained from the ethical review board committee of the Pak Emirates Military Hospital Rawalpindi, Reference No. A/28/60. The sample was obtained via the use of non-probability sequential sampling. Using the WHO sample size calculator and a population prevalence of MS of 0.27 percent, the sample size was determined to be adequate for the study [11].

Inclusion criteria

The research comprised all patients between the ages of 18 and 45 who presented to the neurology outpatient department or were hospitalized to the neurology ward and were diagnosed by the consultant neuro physician with MS or NMO-SD. Other military, public sector, and private sector hospitals that received patients with the same diagnoses as those from their hospital wards were included in the study as well. This disease was diagnosed by combining evidence of damage to the central nervous system (CNS) in at least two locations, such as the brain and spinal cord, as well as the optic nerves, and by determining that the damaged areas appeared at least one month apart. This excluded all other diagnoses and allowed doctors to focus on MS, which was then confirmed with an MRI and a spinal tap for the presence of oligoclonal bands [12].

An AQP4 antibody-positive case of NMOSD must include clinical symptoms and/or MRI abnormalities related to the optic nerve, spinal cord, brain stem, or cerebral presentations to be considered for diagnostic consideration [13]. By combining the findings of several serological tests, the 12NMO-SD strain was identified (NMOSD with or without AQP4-IgG).

Exclusion criteria

Patients under the age of 18 or with an uncertain neurological diagnosis were excluded. Patients who were pregnant had a positive CSF India ink stain for the fungus or were suspected of having Tuberculous meningitis (TBM) or encephalitis were excluded from the research as well. Those suffering poliomyelitis, post-traumatic meningitis, or post-injection syndrome were prohibited from participating. Those with diabetes or neoplasia, hypothyroidism, renal failure, vasculitis, or a history of intoxication were also excluded from the study, as were those who did not give written informed consent or who did not have these conditions.

Data collection

In the neurology unit of MH RWP, individuals with MS or NMO-SD who met the aforementioned criteria for inclusion and exclusion were included in the research after providing written informed permission. Patients with MS were categorised into the clinical subtypes during their stay at the hospital by taking a detailed history, reviewing the charts, and liaising with other treating teams. All the symptoms at the time of presentation along with the systems involved were also documented in the study performa.

Statistical analysis

The Statistics Package for Social Sciences version 24.0 was used for all statistical analyses (SPSS-23.0). The ages of the research participants were averaged and standard deviations computed. Patients with MS and NMO-SD, kinds of MS, and symptoms were reported by gender, frequency, and percentages. Chi-square and regression analysis with p-value<0.05 as significant were applied to look for correlation for various variables with involvement of more than one system at the time of presentation.

## Results

The final study comprised 80 patients, of whom 31 (38.5 percent) were men and 49 (61.25%) were women. The study's MS and NMO-SD patients had a median age of 31.93 years, with a range of 8.97 years to almost 32 years. Table 1 shows the basic characteristics and clinical spectrum of study participants. Fifty-four (67.5%) patients were diagnosed with multiple sclerosis while 26 (32.5%) had neuromyelitis optica spectrum disorders.

**Table 1:** Characteristics and frequency of patients admitted with multiple sclerosis and neuromyelitis optica spectrum disorders

Age (years) Frequency (n=%)	
Mean +/- SD	31.93 ±8.97 years
Range (min-max)	12 years-59 years
Gender	
Male	31 (38.75%)
Female	49 (61.25%)
Clinical Features at Presentation	
Motor	19 (41.3%)
Sensory	29 (63.0%)
Sphincter disturbances	08 (17.4%)
Ataxia	06 (13.1%)
Facial weakness	06 (13.1%)
Vertigo	01 (2.1%)
Visual symptoms	28 (60.8%)
dysarthria	02 (4.3%)
dysphagia	02 (4.3%)
hearing loss	02 (4.3%)
memory problems	05 (10.9%)
psychiatric symptoms	09 (19.5%)
Fatigue	06 (13.1%)
Others	03 (6.5%)
Type of disease	
Multiple sclerosis	54 (67.5%)
Neuromyelitis optica spectrum disorders	26 (32.5%)
Variants of Multiple Sclerosis	
First presentation	08 (17.4%)
Relapsing-remitting	28 (60.9%)
Primary progressive	05 (10.8%)
Secondary progressive	03 (6.5%)
Progressive-relapsing	02 (4.3%)

Most of the patients presented with sensory symptoms followed by visual disturbances and motor symptoms. Twenty-nine (63%) patients had involvement of more than one system, while 51 (37%) patients had involvement of only one system at the time of presentation. Table 2 shows results of Pearson chi-square revealed that female gender and having a diagnosis of multiple sclerosis had a statistically significant relationship with involvement of one than one system (p-value<0.05).

**Table 2:** Relationship of various factors with involvement of more than one system at the time of presentation denoting the frequency of patients

	Frequency of Involvement of one system only n(%)	Frequency of Involvement of more than one system n(%)	P-Value
Age			
35 years or less	38 (74.5%)	18 (62.1%)	0.247
>35 years	13 (25.5%)	11 (37.9%)	
Gender			
Male	29 (56.8%)	2 (6.9%)	<0.001
Female	22 (43.2%)	27 (93.1%)	
Type of Disease			
Multiple sclerosis	24 (47.1%)	2 (6.9%)	<0.001
Neuromyelitis optica spectrum disorder	27 (52.9%)	27 (93.1%)	
Duration of illness			
<3 years	34 (66.7%)	15 (51.7%)	0.189
>3 years	17 (33.3%)	14 (48.3%)	

Binary logistic regression analysis confirmed this association as shown in Table 3.

**Table 3:** The correlated factors relating to the involvement of more than one system among the patients of multiple sclerosis and neuromyelitis optica spectrum disorders: the binary logistic regression analysis

	p-value	Odds ratio	Confidence interval	
			lower	upper
Age (ref. was 35 years or less)	0.984	0.987	0.276	3.527
Duration of illness (reference was <3 years)	0.410	1.638	2.881	13.453
Type of disease (ref. was neuromyelitis optica spectrum disorder)	0.007	10.393	1.889	57.189
Gender (ref. was male)	0.001	14.435	2.881	72.331

## Discussion

Immune-based disorders make a huge chunk of neurological presentations and need an accurate diagnosis for adequate and timely management. Some of these disorders including multiple sclerosis and neuromyelitis optica have been studied less due to their low incidence, overlapping nature, and variety of symptoms [2,3]. Clinical spectrum and involvement of various symptoms predicting the severity of illness need to be understood in the context of our population to provide a clear road map to clinicians in Pakistan to equip themselves better for these rare but serious disorders. Especially, physicians working at tertiary care hospitals should be aware of the whole spectrum of presentation of these disorders because these centers are usually the last resort for the patients who have already been suffering for quite some time in the process of getting referred and reaching the tertiary care facility [14]. One of its rare variants is tumefactive MS that has a prevalence of 1-3/1000 cases. It is quite challenging to diagnose due to its similar features with central nervous system neoplasms on the findings of MRI [15]. We, therefore, planned this study with the rationale to assess the clinical presentation of multiple sclerosis and neuromyelitis optica spectrum disorder in young patients in our community.

In 2008, Lucchinetti et al. published a study that revolved around the clinical spectrum in patients diagnosed with multiple sclerosis. Their findings were that the relapsing-remitting type was the most common type of MS and motor symptoms were the commonest symptoms reported followed by cognitive symptoms. The frontal lobe was the most common lobe in which lesions were seen on neuroimaging modalities. The difference in results may be because we included both MS and NMO SD patients.

A similar study published by Kilic et al in 2013 [17] concluded that the mean age of onset of symptoms was 29 years and half of the patients at the time of presentation had symptoms confined to one system only. Age was the factor significantly related to the presence of symptoms involving more than one system at the time of presentation. Data in our study reflecting a very different population from that of Kilic et al. was understandably different and the mean age of our patients was 31.93 ±8.97 years and 27% of patients symptoms involving more than one system at the time of presentation.

In 2014, Jarius et al. [18] studied the clinical spectrum, immunological basis, and management options for neuromyelitis optical in detail. Over 90% of NMO patients have a relapsing illness pattern characterized by episodes of ocular neuritis, myelitis, or both, which come and go with no apparent pattern. The remaining 10% of patients will have a monophasic course, which is more often linked to optic neuritis and myelitis. Most of our patients had the relapsing-remitting disease, and around a quarter of them had several systems implicated at the time of presentation. Our study's major drawback is that we examined MS and NMO together since they are both uncommon diseases.

In 2019, Beekman et al. [19] reported the clinical spectrum and patient experience among patients of NMO SD. Numbness and tingling were the most common symptoms reported in their study, followed by difficulty in walking and vision problems.

Limitations

We did not include the radiological spectrum in our study. Rather, we focused on the clinical spectrum and reached the conclusion that the relapsing-remitting type was the most commonly encountered type and the sensory system was the most common to be involved, followed by visual problems

The clinical spectrum of patients in our study was quite similar except that after sensory symptoms, our patients reported visual symptoms more than motor symptoms, which was the limitation of our study. More studies with a larger sample size and longer study duration separating the patients of MS and NMO-SD may generate better results in this context.

## Conclusions

Motor, sensory and visual symptoms were commonly found at the time of presentation among patients of multiple sclerosis and neuromyelitis optica spectrum disorders. Involvement of one than one system at the time of presentation was fairly common among these patients, and females and patients having a diagnosis of multiple sclerosis were more at risk of involvement of more than one system.
